# A mixed-methods study on health learning materials utilization for COVID-19 risk communication and community engagement among health workers in Arsi Zone, Ethiopia: *Implication for response to pandemic*

**DOI:** 10.1371/journal.pone.0269574

**Published:** 2022-06-07

**Authors:** Taye Debele, Firanbon Teshome, Demuma Amdisa, Girma Bacha, Zewdie Birhanu, Yohannes Kebede

**Affiliations:** 1 Arsi University Asella Teaching and Referral Hospital, Asella, Oromia, Ethiopia; 2 Faculty of Public Health, Department of Health, Behavior and Society, Jimma University, Jimma, Ethiopia; 3 School of Nursing, Faculty of Health Science, Jimma University, Jimma, Ethiopia; IGMC: Indira Gandhi Medical College, INDIA

## Abstract

**Background:**

Risk communication and community engagement are among the key strategies used in response to pandemics. Effective risk communication and community engagement can be achieved when assisted by health learning materials. However, their utilization was not known in Ethiopia. Therefore, the present study aimed to assess the utilization of COVID-19 health learning materials (HLMs), and explore barriers and facilitating factors.

**Methods:**

A sequential explanatory mixed-methods study consisting of two phases was carried out. The first phase was a cross-sectional survey to assess the utilization of COVID-19 HLMs and their predictors. In this phase, a multistage sampling technique was used to select 530 health workers. A self-administered structured questionnaire was used for data collection. Epi-data manager version 4.6.0.2 and STATA version 16 were used for data entry and analyses, respectively. Descriptive analyses were carried out as necessary. Ordinal logistic regression analyses were done to identify the predictors of COVID-19 HLMs utilization. Phase two is a qualitative study to explore enablers and barriers to COVID-19 HLMs utilization. A judgmental sampling technique was used and 14 key informants were recruited. The collected data were uploaded into Atlas ti version 7.0.71. An inductive process of thematic analysis was employed and the data were coded, categorized, and thematized.

**Results:**

Findings showed that out of the total 530 respondents, 210(39.6%), 117(22.1%), and 203(38.3%) of them never use COVID-19 HLMs, use sometimes, and always, respectively. Health workers’ perceived quality of COVID-19 HLMs [AOR = 6.44 (95% CI: 4.18–9.94)], health workers’ perceived usefulness of COVID-19 HLMs [AOR = 2.82 (95% CI: 1.88–4.22)], working facility [AOR = 1.83 (95% CI: 1.07–3.14)], educational level of the respondents [AOR = 1.73 (95% CI: 1.11–2.72)] and availability of COVID-19 HLMs [AOR = 1.45(95% CI: 1.01–2.08)] had statistically significant association with the utilization status of COVID-19 HLMs. Findings from the qualitative study showed that materials-related factors, and structure and health workers-related factors had influence on HLMs utilization.

**Conclusions:**

In this study, we found that only a few of the respondents were regularly utilizing COVID-19 HLMs. Perceived quality, usefulness, and availability of HLMs, and health workers’ educational status and working facility determined the level of COVID-19 HLMs utilization. There is a need for giving due attention to HLMs, evaluating their quality, availing them to health facilities, and providing training for health workers.

## Background

Pandemic is a serious public health problem that has several negative consequences such as health, social, political, economic, psychological, educational, religious impacts, and national instability [1–5]. In long term, pandemics have a great impact on life quality [6]. For instance, evidence showed that in 2009–2010, influenza pandemics killed 8,870–18,300 people worldwide [7]. The Ebola outbreak has seriously affected the economy, especially in West Africa [8]. Similarly, the COVID-19 pandemic has spread exponentially and caused enormous crises. For example, a systematic review and meta-analysis from England and France showed that 74.37% of deaths occurred among COVID-19 cases [9]. COVID-19 increased maternal morbidity and mortality, and neonatal complications [10]. Apart from the morbidity and mortality, COVID-19 has had enormous negative impacts on the socio-economic and health systems of the world [11,12]. Indeed, it exacerbates the preexisting education disparities by reducing the opportunities of many individuals to continue their learning [13,14], affecting nearly 1.6 billion learners in more than 190 countries [13].

The world health organization and many countries have been designing different policies, programs, and strategies for the prevention and control of pandemics. Risk communication and community engagement are among some of the key strategies used in response to pandemics [15–17]. Evidence showed that effective risk communication and community engagement have the potential to increase awareness, combat uncertainties, save countless lives, and preserve public health crises such as the economic, political, and social impacts of pandemics [18–25]. These can be achieved when risk communication and community engagement are assisted by health learning materials [26,27].

Health learning materials (HLMs) are the most cost-effective strategies that serve to improve knowledge, perceptions, skills, attitudes, beliefs, and key health behaviors by communicating risks, quickly reaching information, providing detailed facts and raising public awareness, reminding of key messages, reinforcing communications, mobilizing the community, increasing motivation and self-efficacy of the target audiences, counteracting rumors, reducing fears, solving doubts and misconceptions, reducing stigma and discrimination [28–34]. For instance, evidence showed that HLMs played a significant role in the mitigation of infectious diseases [35–37], improving maternal and child health [38,39], and increasing health-seeking behavior [40,41]. Even though HLMs are recommended and have numerous contributions to the prevention and control of pandemics and other public health emergencies [26,27], to the best of the authors’ knowledge, their utilization status in response to the COVID-19 pandemic was unknown yet. Therefore, this study aimed to assess the utilization status of COVID-19 HLMs and explore their enabling and hindering factors among health workers.

## Objectives

To assess utilization of COVID-19 health learning materials and its predicting factors among health workers in Arsi zone, Oromia, Ethiopia, 2021.

To explore facilitators and barriers related to COVID-19 health learning materials utilization among health workers in Arsi zone, Oromia, Ethiopia, 2021.

## Methods

### Study area and period

The study was conducted from May 15 to June 25, 2021, in the Arsi zone. The Arsi Zone is one of the 20 zones found in Oromia Regional State, Southeast Ethiopia. The capital of the Arsi zone is Asella town, which is located about 175 km southeast of Addis Ababa. According to the 2021 report obtained from the Arsi Zone health office, the Zone has a total population of 3.5 million. The zone has 26 districts with five primary hospitals, one specialized teaching hospital, and 106 primary health care units (PHCUs) that provide disease prevention and health promotion services. A total of 2,032 health workers of different professions were found in the zone.

### Study design

In this study, a sequential explanatory mixed-method study design was used and both qualitative and quantitative data were collected, analyzed, and integrated. The sequential explanatory design is a mixed method that firstly collects and analyses quantitative data, and then qualitative data to explain and/or generalize quantitative data which leads to a better understanding of the problem [42,43]. In phase one of this study, we first conducted a cross-sectional study to determine the level of health workers’ COVID-19 HLMs utilization and its predictors. In the second phase, an exploratory qualitative study was conducted for a deeper explanation of COVID-19 HLMs utilization, facilitating, and inhibiting factors. In phases one and two of the study, the methods were described in detail.

## Phase one: A quantitative study

This phase was a cross-sectional survey, aimed to determine HLMs utilization and associated factors among health workers in Arsi Zone, Southeast Ethiopia, 2021.

### Population

Source populations were all health workers of the Arsi zone. The study populations were all randomly selected health workers in the study area. All health workers who were working in the zone during the study period were included in the study. Health workers who were severely ill and did not exist in the zone due to different reasons during the data collection period were excluded.

### Sample size and sampling technique

The sample size in the quantitative phase was calculated using a single population proportion formula with the assumption of 50% proportion of health learning materials since there were no studies in Ethiopia that can address these objectives, 1.96 standard normal distribution curve value for 95% level of confidence and 5% margin of error between the sample and the population. Using the formula, the sample size becomes 384. Since the source population is less than 10,000, the corrective formula was used to calculate the sample size and it becomes 323. After considering a 10% non-response rate and 1.5 design effects, the final sample size becomes 534.

A multistage sampling technique was used to select the study participants as follows: In the first stage, among the 26 districts in the Arsi Zone, 8 districts were selected by a simple random sampling technique. Then, the health facilities were stratified into hospitals and primary health care units (health centers and health posts). In the second stage, four hospitals and four primary health care units (PHCUs) were selected using a simple random sampling technique. Then, the sample size was proportionally allocated to the selected 8 health facilities. Accordingly, a total of 534 health workers (Asela Hospital = 210, Sude Hospital = 54, Golja PHCU = 22, Dera PHCU = 22, Bekoji Hospital = 70, Bale PHCU = 18, Robe Hospital = 115, Ogolcho PHCU = 23) were selected to take part in the study. The list of health workers was obtained from the master head of respective district health offices. Accordingly, a sampling frame of 620 health workers was constructed. Finally, a computer-generated simple random sampling technique was employed to identify the study participants.

### Data collection tools and procedures

For the quantitative data collection, a structured questionnaire was adapted from relevant literature [32,44–49]. The questionnaire was initially prepared in English and then translated to the local languages (Afaan Oromoo and Amharic) by a master holder translator who is a fluent speaker of the languages. Thereafter, it was back-translated to the English language by another translator. The questionnaire comprises four sections that sought information on socio-economic and demographic characteristics, availability and utilization of HLMs, perceived usefulness, and perceived quality of HLMs for COVID-19 risk communication and community engagement. Utilization of COVID-19 HLMs was assessed using one question (*Do you use COVID-19 HLMs for risk communication and community engagement*?) which was scored on a three-point Likert scale with response options “1 = never, 2 = sometimes, and 3 = always”. Health workers’ perceived usefulness of COVID-19 HLMs was measured using 16 items (*e*.*g*. *I believe that COVID-19 HLMs are helpful reminders for key messages about the pandemic*) on a five-point Likert scale with response options “1 = strongly disagree, 2 = disagree, 3 = undecided, 4 = agree, and 5 = strongly agree”. Health workers’ perceived quality of COVID-19 HLMs was measured using 29 items (*e*.*g*. *Messages of COVID_19 HLMs are fact-based and up-to-date information*) on a five-point Likert scale with response options “1 = strongly disagree, 2 = disagree, 3 = undecided, 4 = agree and 5 = strongly agree”.

A pretest was conducted on 5% of the total sample size in Shashamane Hospital and Dodola PHCU, which are located 70 km and 35 km away from the study area, respectively. Some modifications were done based on the findings. The internal consistency of the items was tested using Cronbach’s alpha. Accordingly, the alpha of the perceived quality of HLMs was 0.929, and the perceived usefulness of HLMs was 0.935, which was within the acceptable range [50,51]. The validity of the questionnaire was checked by different experts [50,51].

The data were collected through self-administered interviews. A total of 8 supervisors were recruited based on their previous experience in data collection and fluency in the local languages. Intensive training was given to the supervisors on the objective of the study, the data collection tools and procedures, approaches to the study participants, and respecting and maintaining the confidentiality of the respondents. As health workers were busy with serving clients, they were requested to fill out the questionnaire while they get free time which beats the health facility, waiting area, ward, unit, and staff cafeteria.

### Variables

The dependent variable was the level of COVID-19 HLMs utilization. The independent variables were socio-economic and demographic factors (age, sex, residency, religion, ethnicity, marital status, professional category, educational level, working facility and unit, work experience, and salary), HLMs related factors such as availability of COVD-19 HLMs, health workers perceived quality and usefulness of COVD-19 HLMs.

### Operational definitions and measurements

***Health workers*:** In the current study, health workers included medical doctors, nurses, public health officers, druggists or pharmacists, laboratory technicians or technologists, and health extension workers.

***Health learning materials (HLMs)*:** In this study, HLMs included printed media (posters, brochures, flyers, flipcharts, banners, stickers, and newsletters), audio, and audio-visual messages, and materials found in health facilities for COVID-19 risk communication and community engagement.

***HLMs Utilization***: In this study, the COVID-19 HLMs utilization status of health workers was ordered as never use COVID-19 HLMs, use them sometimes, and use them always(regularly) for the sake of risk communication and community engagement during the pandemic.

***Perceived quality*:** Health workers’ perceptions about the comprehensiveness, attractiveness, acceptability of the materials, involvement of target audiences, and materials’ ability to call the target audiences to carry out a particular action. In this study, the perceived quality of COVID-19 HLMs was measured by 29 items. The negatively worded items were reverse scored. Then, the composite perceived quality score was computed by summing all the 29 items. Finally, dichotomization was made by taking the mean score as a cut point. Scores above or equal to the mean (≥81.75) were considered as ‘*COVID-19 HLMs have quality*’ and scores below the mean (<81.75) were considered as ‘*COVID-19 HLMs have no quality’*.

***Perceived usefulness*:** Health workers’ perceptions about the benefits and roles of COVID-19 HLMs such as communicating risks, supplementing verbal messages, providing detailed facts and raising public awareness, increasing the self-efficacy of the target audiences, reducing rumors, fears, doubts, misconceptions, stigma, and discrimination related to the pandemic, serving as reminders for key messages, stimulating the community and helping them to comply with the COVID-19 preventive and control measures. In this study, the perceived quality of COVID-19 HLMs was measured by 16 items. The negatively worded items were reverse scored. Then, the composite perceived usefulness score was computed by summing all 16 items. Finally, dichotomization was made by taking the mean score as a cut point. Scores above or equal to the mean (≥45.46) were considered as ‘*COVID-19 HLMs are useful*’ and scores below the mean (<45.46) were considered as ‘*COVID-19 HLMs are not useful*’.

***Availability of HLMs*:** In this study, the COVID-19 HLMs were considered available if at least one type of HLMs were available in the health facility and health care providers were able to access it while they need to teach about COVID-19.

***The primary health care unit (PHCU****)*: is the smallest division in the Ethiopian health tier system which usually includes one health center and five health posts.

### Data quality control

A pretested questionnaire was used for data collection. The tool was translated to local languages considering the significant difference in the educational level of the study participants. Intensive training was given to supervisors and the data collection was closely supervised. The internal consistency and some dimensions of validity of the tool were assured by experts.

### Data analysis

The quantitative data were entered into Epi-data manager version 4.6.0.2 software. Then, the data was transported to STATA version 16 for analyses and cleaning. In this study, outliers were identified based on the p-value. Descriptive statistics such as frequency, percentage, mean and standard deviation were done to describe the study participants in relation to relevant variables. Since the outcome variable was the ordinal type of categorical data, univariable and multivariable ordinal logistic regression analyses were carried out to test the association of COVID-19 HLMs utilization with explanatory variables. Crude and adjusted proportional odds ratio and 95% CI were used to determine the magnitude of the association. Variables that have a p-value <0.25 in the univariable ordinal regression were fitted into the multivariable ordinal logistic regression model to control the effect of confounding variables and identify predictors of COVID-19 HLMs utilization. The level of statistical significance was set at a p-value of < 0.05. Assumptions of ordinal logistic regression analyses were checked by likelihood ratio test, goodness-of-fit, and test of parallel lines before the attempt to interpret the final model, and no violation was sought. Finally, results were presented in the form of tables, figures, and narratives.

## Phase two: A qualitative study

### Study design and setting

A descriptive qualitative study was conducted following the quantitative study to explore enablers and barriers to the utilization of HLMs in Arsi zone, Oromia, Ethiopia. Health workers from hospitals, health centers, and health posts were included in the qualitative study. The study setting was detailed in phase one.

### Population and sampling approach

A judgmental sampling technique was used in this phase and diversified key informants were recruited considering their educational level, professional category, work experience, working facility, and their specific working units. The author contacted key informants to recruit them, introduced himself, and clarified the reasons for the study before starting data collection. Accordingly, a total of 14 key informants (2 health extension workers, 2 nurses, 3 public health officers, 1 environmental health, and 6 specialties in public health masters) participated in the study. All the recruited informants participated in the study. In terms of educational level, 3 key informants were diploma holders, 5 degrees, and 6 master holders (Table 1).

**Table 1:** Summary of background characteristics of the key informants.

Variable	Category	N	Variable	Category	N
Age(yrs.)	30–34	7	Working facility	Health post	2
	35–39	5		Health center	2
	≥40	2		Hospital	5
Sex	Male	9		District health office	2
	Female	5		Zonal health office	3
Educational level	Diploma	3	Work experience	<5 yrs.	1
	Degree	5		5–10 yrs.	9
	Masters	6		>10 yrs.	4
Professional category	Health extension	2	Specific working unit	PHEM	2
	Nurse	2		Maternal and child health	1
	Health officer	3		CDC	4
	Env’tal health	1		Env’tal health	5
	Specialty (MPH)	6		Health post	2

CDC: Communicable disease control; Env’tal: Environmental; PHEM: Public health emergency management.

### Data collection

A semi-structured interview guide with open questions was prepared by the authors based on the research questions. In addition, outliers identified in the survey were fed into the development of the interview guide. The guide was first prepared in the English language. Then, it was translated to the local language and back-translated to English to ensure its consistency and accuracy. The interview guide has four main parts: A) General questions about HLMs (e.g. *would you tell me any health learning materials you know*? *Probe*: *Can be printed Media*? *Audio or audiovisuals*? *Folk media*?*)* B) Availability and utilization of COVID-19 HLMs (e.g. *Do the health workers are using COVID-19 HLMs for risk communication and community engagement*? *Probe*: *Types*?*)* C) Perceived quality of COVID-19 HLMs (e.*g*. *How do you think the quality of COVID-19 health learning materials*? *Probe*: *Clarity and simplicity of messages*? *Eye-catching*? *Inviting illustrations and texts*? *Call to action*?*)*, and D) Perceived usefulness of COVID-19 HLMs (*e*.*g*. *How do you perceive the usefulness of health learning materials*? *Probe*: *Solving rumors*? *Communicating risks*? *Engaging population*? *Encouraging appropriate behaviors*?). The recruitment of key informants was held five days before data collection. The date of the interview, time, and area was decided after discussion with the key informants. On the day of the interview, the investigator established good rapport, explained the aim of the study, and obtained consent from the informants. Face-to-face interviews were conducted in the workplace of key informants (in private rooms) in a way that maintains their confidentiality. Voices were recorded for all interviews. In addition, the interviewer also took note of verbal and non-verbal communication such as tone of voice and facial expressions. The interviews were averagely run for 34 minutes. In order to keep the consistency, all the interviews were conducted by one author. The data were collected until saturation was achieved.

### Data analysis

First, transcription was made word by word for all the audio tape-recorded data. The transcripts were translated from local languages to the English language. The translated data were uploaded into Atlas ti version 7.0.71 software. The authors read and reread all the transcripts to obtain a full understanding of them. An inductive process of thematic analysis was employed and the data were coded line by line. Two authors participated in coding the data. For codes where the coding conflict occurred, an agreement on their meaning and interpretation was reached through discussion. Finally, the codes were clustered into meaningful groups to form categories and themes. Concepts were extracted from themes and presented in narratives, and direct quotes were selected to illustrate the interpretations.

### Trustworthiness

In this study, we checked the credibility, dependability, transferability, and conformability of the findings using different techniques. Triangulation of data was made by involving diversified key informants in the study. The interviewer built a relationship with the key informants by greeting and welcoming them, injecting some elements of humor in the conversation, discussing the aim of the study with the participants, starting the interviews with general and light questions for icebreaking, and gradually moving to specify questions, actively listening to them through verbal cues (aha…right. ok) and non-verbal encouragers (eye contact, nodding head), and showing sincere interest in receiving their ideas. Qualitative experts and colleagues were involved in the analysis for auditing and verifying the findings and their interpretations. Each interview was summarized at the end session of key informant interviews. Indeed, interviewees were asked to check whether the summaries were their own ideas and requested to provide their feedback. The data collector stayed in the field all during the recruitment of the key informants and the data collection period. Each transcript was checked against audio-recorded data and field notes. All the research processes and methodological approaches were explained in detail which helps to ensure the thick description of the study and enables readers to understand how the authors reached study conclusions. Indeed, the chronology of research processes, analysis, and the findings was audited and validated by researchers who had rich experience of interviewing and qualitative data management skills. As best as possible, the authors intended only to answer the research question of the study with minimal interpretation bias. To ensure this, different people were involved in data coding, interviews were summarized at the end of each interviewing session, participants were asked for feedback, and authors audited and verified the research processes, audio-record data against transcripts, and analysis and interpretation of the findings. Furthermore, researchers’ reflexivity practices in this study were detailed as follows: Five out of the six authors were public health experts who had health education and promotion, and/or health behavior and communication educational background that have rich experience in the areas of HLMs, risk communication, community engagement, and social and behavioral change communication. One author is nursing in his educational background. Two of the authors are Ph.D. holders, one is a Ph.D. fellow and three authors had master’s educational levels. Five out of the six researchers are male. The authors have ample experience in conducting quantitative, qualitative, and mixed-method studies.

### Ethical approval

Ethical clearance was obtained from the Research and Ethical Review Committee of Jimma University with the reference number: IHR-PGH/201/21. In addition, a permission letter was secured from the Arsi zone and district health offices. Written informed consent was taken from each study participant. All participants were informed about the objectives of the study. Furthermore, the participants were reassured that their responses were kept confidential, and anonymous.

### Schematic presentation of methods

**Fig 1:**
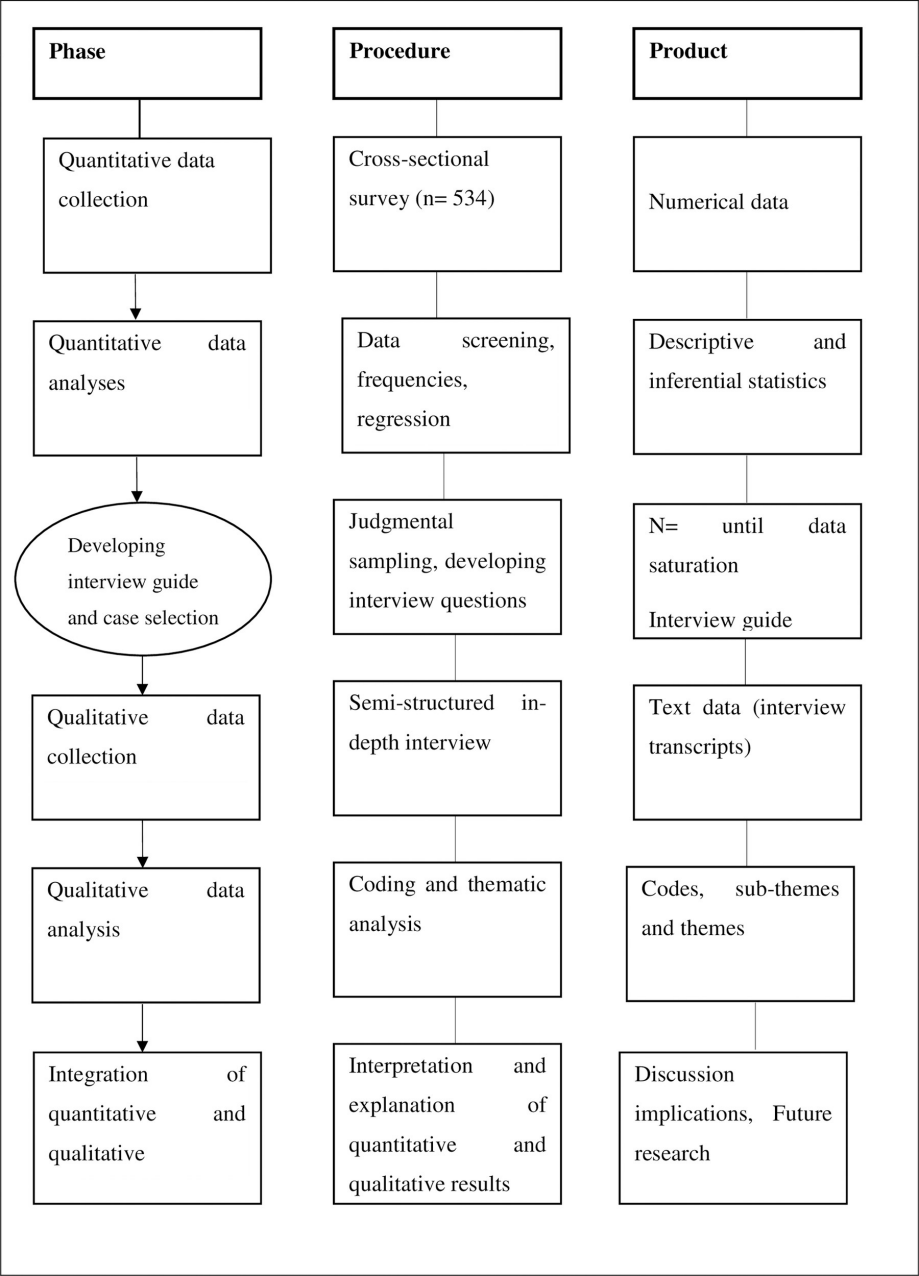
Methods structure diagram.

The main contents of the methods section are explained in a diagram (Fig 1).

## Results

### Socio-economic and demographic characteristics of the respondents

A total of 530 health workers participated, giving a response rate of 99.3%. The mean age of the respondents was 37.55(±5.65). Findings showed that most of the respondents, 486 (91.7%), 447 (84.3%), and 415 (78.3%) were urban residents, clinicians, and working in hospitals, respectively. More than three fourths (75.7%) had an educational status of degree and above. The majority of the respondents, 417 (78.7%) had work experience of greater than five years (Table 2).

**Table 2:** Socio-economic and demographic characteristics of health workers in Arsi Zone, Oromia, southeast Ethiopia, 2021 (N = 530).

Variable	Category	Frequency	Percent
Age	Mean age	37.55(±5.65)	
Professional category	Health extension worker	44	8.3
	Medical doctor	39	7.4
	Nurse	237	44.7
	Public health officer	60	11.3
	Druggist or Pharmacist	83	15.7
	Lab. Technician/technologist	67	12.6
Types of health facility	Hospital	415	78.3
	Health center	71	13.4
	Health post	44	8.3
Residency	Rural	44	8.3
	Urban	486	91.7
Educational level	Diploma	129	24.3
	Degree and above	401	75.7
Sex	Female	240	45.3
	Male	290	54.7
Religion	Orthodox	202	38.1
	Muslim	240	45.3
	Protestant	56	10.6
	Wakefata	32	6.0
Ethnicity	Oromo	373	70.4
	Amhara	141	26.6
	Other	16	3.0
Marital status	Single	141	26.6
	Married	337	63.6
	Divorced	25	4.7
	Engaged	27	5.1
Work experience	<5 yrs.	113	21.3
	≥5 yrs.	417	78.7
Working unit	Working in a chronic unit	42	7.9
	Working in a non-chronic unit	488	92.1
Salary (ETB)	6000–7999	122	23.0
	8000–9999	285	53.8
	> = 10000	123	23.2

### COVID-19 HLMs related factors

In this study, only 55.7% of the respondents responded that COVID-19 HLMs were available in their health facilities. Nearly half, 231(43.6%) and 246(46.4%) perceived that COVID-19 HLMs had no quality and were not useful for risk communication and community engagement (Table 3).

**Table 3:** Factors related to COVID-19 health learning materials in Arsi Zone, Oromia, southeast Ethiopia, 2021 (N = 530).

Variable	Category	Frequency	Percent
Availability of COVD-19 HLMs	Unavailable	235	44.3
	Available	295	55.7
Perceived quality of COVD-19 HLMs	Have no quality	231	43.6
	Have quality	299	56.4
Perceived usefulness of COVD-19 HLMs	Not useful	246	46.4
	Useful	284	53.6

#### Level of COVID-19 HLMs utilization

**Fig 2:**
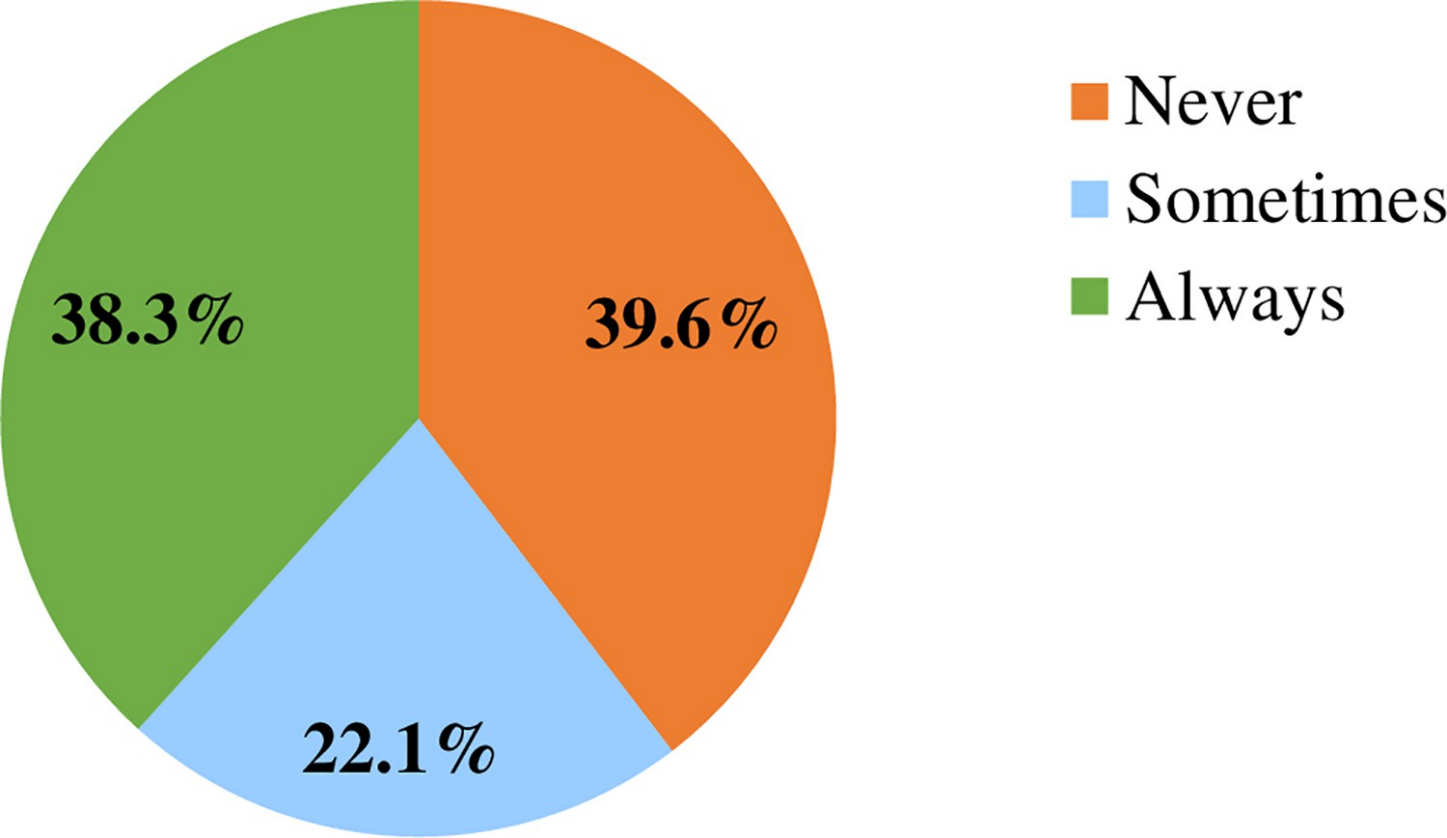
Level COVID-19 health learning materials utilization among health workers in Arsi Zone, Oromia, southeast Ethiopia, 2021 (N = 530).

In this study, out of the total 530 respondents, 210 (39.6%) of them never use COVID-19 HLMs for risk communication and community engagement. Findings showed that only 203 (38.3%) of the respondents always use COVID-19 HLMs for risk communication and community engagement. Nearly one-fifth, 117 (22.1%) of the respondents sometimes use COVID-19 HLMs for risk communication and community engagement (Fig 2).

#### COVID-19 HLMs utilization contexts

**Fig 3:**
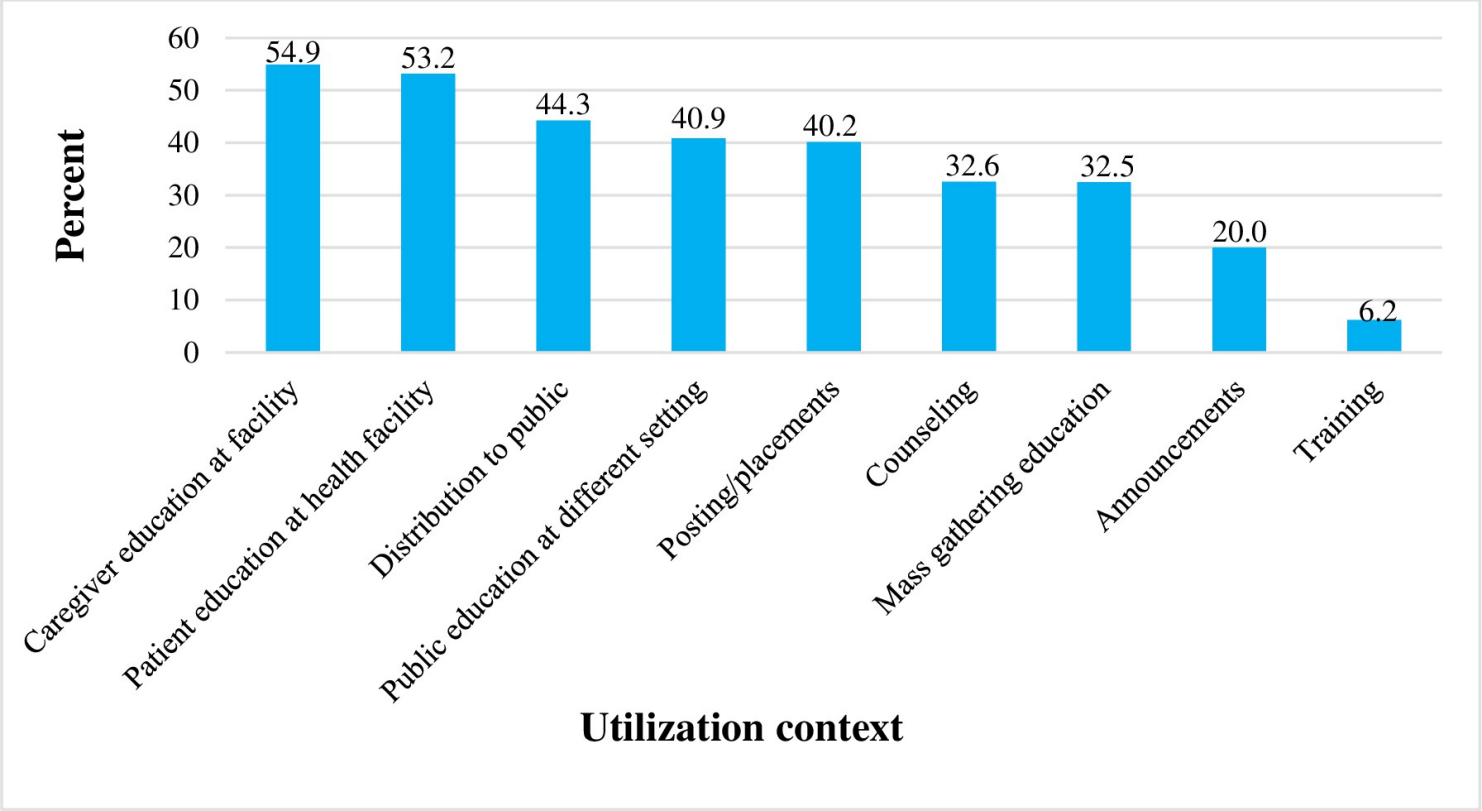
Contexts of COVID-19 HLMs utilization among health workers in Arsi Zone, Oromia, southeast Ethiopia, 2021 (N = 530).

In this study, out of 530 respondents, nearly half, 291 (54.9%) and 282 (53.2%) of them utilize COVID-19 HLMs for caregivers and patients education at health facilities, respectively. One-fifth, 106 (20.0%) of the respondents utilize COVID-19 HLMs for announcements. Only 33 (6.2%) of the respondents utilize COVID-19 HLMs while providing training on the COVID-19 pandemic (Fig 3).

#### Assumptions of ordinal logistic regression

The inter-correlations among independent variables were diagnosed using the variance inflation factor (VIF). In this study, the maximum VIF was 1.56 which was less than five [52] indicating that there was no multi-collinearity among the predictor variables. Findings from the crosstab between predictors and the outcome variable showed that none of the cells was too small or empty, which indicates that the model was stable and fit to run. A likelihood ratio chi-square test from model fitting information showed that there was a significant improvement in the fit of the final model over the null model (X2 = 200.088, P-value< 0.001). The non-significant test results of the Pearson chi-square test (X^2^ = 562.873, P = 0.204) and Deviance chi-square test (X2 = 551.626, P = 0.311) suggested that a model exhibits a good fit to the data. The result from a test of parallel lines was non-significant (P = 0.830) indicating that the assumption was satisfied (Table 4).

**Table 4:** Assumptions of ordinal logistic regression (N = 530).

Index	Value
Likelihood Ratio Test	X^2^ = 200.088, P-value< 0.001
Goodness-of-Fit	Pearson X^2^ = 562.873, P-value = 0.204
	Deviance X^2^ = 551.626, P-value = 0.311
Test of parallel lines	X^2^ = 7.402, P-value = 0.830

### Factors associated with COVID-19 HLMs utilization

#### A) Univariable ordinal logistic regression analysis

Table 5 shows univariable ordinal logistic regression analysis. Findings indicated that a total of ten variables (age of the respondents, type of health facility, residency, educational level of the respondents, work experience, risk communication training, availability of COVID-19 HLMs, perceived quality of HLMs, perceived usefulness of HLMs, and monthly salary) had a statistically significant association with COVID-19 HLMs utilization status (Table 5). In Univariable ordinal logistic regression analysis, perceived quality of COVID-19 HLMs [COR = 9.09 (95% CI: 6.27–13.18)], Perceived usefulness of COVID-19 HLMs [COR = 5.62 (95% CI: 3.97–7.96)], residency of respondents [COR = 2.50 (95% CI: 1.38–4.52)] and availability of COVID-19 HLMs [COR = 1.60 (95% CI: 1.16–2.20)] showed significant association with COVID-19 HLMs utilization at P-value of < 0.01.Type of health facility [COR = 1.60 (95% CI: 1.09–2.33)] and age of the respondents ≥41 years [COR = 0.59 (95% CI: 0.39–0.90)] had statistically significant association with COVID-19 HLMs utilization at P-value of < 0.05. All variables in univariable ordinal logistic regression analysis had a P-value of <0.25 (Table 5).

**Table 5:** Univariable ordinal logistic regression analysis of COVID-19 HLMs utilization among health workers in Arsi zone, Oromia, Ethiopia, 2021 (N = 530).

Variable	Category	COVID-19 Utilization HLMs			cPOR [95% CI]	P-value
		Never	Sometimes	Always		
Age(yrs.)	≤35	80	37	102	Ref	
	36–40	76	52	64	0.71[0.49–1.01]	0.059
	≥41	54	28	37	0.59[0.39–0.90]	0.015
Type of health facility	Hospital	178	85	152	1.60[1.09–2.33]	0.016
	PHCU	32	32	51	Ref	
Residency	Rural	8	11	25	2.50[1.38–4.52]	0.002
	Urban	202	106	178	Ref	
Educational level	Diploma	50	22	57	1.21[0.83–1.76]	0.215
	≥ Degree	160	95	146	Ref	
Risk communication training	Yes	147	100	158	1.47[0.99–2.15]	0.051
	No	63	17	45	Ref	
Availability of COVID-19 HLMs	Available	138	52	105	1.60[1.16–2.20]	0.004
	Unavailable	72	65	98	Ref	
Perceived quality of HLMs	Have quality	49	85	165	9.09[6.27–13.18]	<0.001
	Have no quality	161	32	38	Ref	
Perceived usefulness of HLMs	Useful	54	80	150	5.62[3.97–7.96]	<0.001
	Not useful	156	37	53	Ref	
Work experience	<5 yrs.	54	13	46	Ref	
	≥5 yrs.	156	104	157	1.23[0.79–3.76]	0.210
Salary(ETB)	6000–7999	40	35	47	Ref	
	8000–9999	109	62	114	0.93[0.63–1.36]	0.694
	≥10000	61	20	42	0.63[0.40–1.01]	0.055

#### B) Multivariable ordinal logistic regression analysis

Table 6 presents the findings of multivariable ordinal logistic regression analysis. Even after adjusting for confounding variables; the types of health facility, educational level of the respondents, availability of COVID-19 HLMs, health workers’ perceived quality, and usefulness of COVID-19 HLMs remain to have a statistically significant association with the utilization status of COVID-19 HLMs. The findings showed that health workers who perceived the quality of COVID-19 HLMs were nearly six times [AOR = 6.44 (95% CI: 4.18–9.94)] at a higher level of COVID-19 HLMs utilization for risk communication and community engagement compared to those who perceived that COVID-19 HLMs had no quality. The perceived usefulness of HLMs also influenced their utilization. Health workers who perceived the usefulness of COVID-19 HLMs were nearly three times [AOR = 2.82 (95% CI: 1.88–4.22)] at a higher level of COVID-19 HLMs utilization for risk communication and community engagement compared to those who perceived that COVID-19 HLMs hadn’t been useful. Findings showed that health workers who were working in hospitals were nearly two times [AOR = 1.83 (95% CI: 1.07–3.14)] at a higher category of utilizing COVID-19 HLMs compared to those who were working in PHCU (health centers or health posts). In this study, the educational level of the respondents also predicted COVID-19 HLMs utilization. Those who had a Diploma educational level were nearly two times [AOR = 1.73 (95% CI: 1.11–2.72)] at a higher level of utilizing COVID-19 HLMs compared to their counterparts. The availability of HLMs had also determined their utilization. The COVID-19 HLMs were nearly 1.5 times [AOR = 1.45 (95% CI: 1.01–2.08)] at higher utilization levels in areas where they were available compared to in areas where COVID-19 HLMs were unavailable (Table 6).

**Table 6:** Multivariable ordinal logistic regression analysis of COVID-19 HLMs utilization among health workers in Arsi zone, Oromia, Ethiopia, 2021 (N = 530).

Variable	Category	COVID-19 Utilization HLMs			aPOR [95% CI]	P-value
		Never	Sometimes	Always		
Age(yrs.)	≤35	80	37	102	Ref	
	36–40	76	52	64	0.73[0.48–1.11]	0.134
	≥41	54	28	37	0.63[0.38–1.05]	0.072
Types of health facility	Hospital	178	85	152	1.83[1.07–3.14]	0.027
	PHCU	32	32	51	Ref	
Residency	Rural	8	11	25	1.71[0.80–3.68]	0.168
	Urban	202	106	178	Ref	
Educational level	Diploma	50	22	57	1.73[1.11–2.72]	0.016
	≥ Degree	160	95	146	Ref	
Training on risk communication	Yes	147	100	158	1.06[0.69–1.64]	0.783
	No	63	17	45	Ref	
Availability of COVID-19 HLMs	Available	138	52	105	1.45[1.01–2.08]	0.045
	Unavailable	72	65	98	Ref	
Perceived quality of HLMs	Have quality	49	85	165	6.44[4.18–9.94]	<0.001
	Have no quality	161	32	38	Ref	
Perceived usefulness of HLMs	Useful	54	80	150	2.82[1.88–4.22]	<0.001
	Not useful	156	37	53	Ref	
Work experience	<5.yrs.	54	13	46	Ref	
	≥5.yrs.	156	104	157	1.14[0.71–1.84]	0.582
Monthly salary (ETB)	6000–7999	40	35	47	Ref	
	8000–9999	109	62	114	0.92[0.59–1.42]	0.701
	≥10000	61	20	42	0.79[0.47–1.34]	0.381

#### Facilitators and barriers related to COVID-19 HLMs utilization

The findings of this study were thematized into two major areas: Materials related factors, and structure and health workers-related factors.

#### Materials related factors

This theme has three categories which include availability and adequacy of COVID-19 HLMs, perceived quality, and usefulness of HLMs.

*Availability and adequacy of COVID-19 HLMs*. The majority of key informants explained that COVID-19 HLMs were usually unavailable to utilize. However, some of them mentioned that they had audiovisual and printed HLMs such as posters, brochures, and leaflets. The study participants pointed out that even in areas where HLMs were available, they were not enough to utilize as per need. The study participants repeatedly mentioned that the unavailability and inadequacy of COVID 19 HLMs can be due to the lack of production of HLMs at the zonal level.

*“…I have only a poster at hand. Since I do not have other HLMs*, *our community has become familiar with this poster. Hence I am not regularly using it” (Rural, HEW)*

*“…In addition to printed COVID-19 HLMs, we have TV shows that broadcast news, panel discussions, public speech announcements, films, short dramas, comedy shows, and music video spots on COVID-19 at some units such as Diabetes, ART, Fistula, and MCH unit*. *However, there are scarcities to avail them at all units. This can be due to lack of HLMs production facility in Arsi zone “(Hospital COVID-19 focal person)*

*Perceived quality of HLMs*. The majority of the key informants repeatedly mentioned that COVID-19 HLMs and messages are unclear and not easily understandable by target audiences, unattractive, culturally unacceptable, and do not represent the community of the study area. The study participants mentioned that this could be due to a lack of engaging communities during the development of materials. They also pointed out that the poor quality of HLMs and messages may be due to a lack of pretesting draft materials among primary target audiences. However, the majority of them mentioned that COVID-19 HLMs call the target audiences to do or not to do a particular action.

“*The language needs to be clear and easy to understand*, *without ambiguities*, *abbreviations*, *jargon*, *and medical terminology*. *For example*, *‘Wal bukkee hin dhaabatinaa’ ordering to ‘keep physical distance’ has more than two meanings and sensitive in Arsi zone"(Rural*, *HEW)*

"*… ..Yeah, the majority of the photographs, graphics, illumination, and animations in HLMs don’t represent our community’s local cultural background*. *For example, where is ethnic clothing such as ‘calle’, ‘cico’, and ‘sinqee’ for mother and ‘head towel’ and ‘boku’ for father? They do not reflect the ethnic and cultural background of the target audience. They are downloaded from the internet which is difficult to understand especially for rural communities where the majority are uneducated "(PHCU COVID-19 focal person)*.

*“COVID-19 HLMs can convey messages that increase awareness, inform benefits of taking COVID-19 preventive measures such as hand washing*, *keeping physical, distance, wearing masks” (District RRT member, 34-year-old)*.

*Perceived usefulness of COVID-19 HLMs*. The majority of the study participants perceived that the COVID-19 HLMs had no significant role in the prevention and control of the pandemic. They mentioned that the majority of the materials were poor at communicating risks, solving misconceptions, supplementing verbal communications, stimulating communities, and encouraging target audiences to comply with COVID-19 preventive and control measures. However, some of the study participants perceived that COVID-19 HLMs had a substantial contribution in raising public awareness and the quick reach of information on COVID-19 preventive measures.

*“I do not think COVID-19 HLMs help to solve rumors and misconceptions related to the pandemic*. *The majority of the pictures are not life pictures and I do not believe the materials can be able to motivate people to comply with the preventive measures” (District, rapid response team focal person)*.

*"… .There are posters posted at public gathering areas such as at market, car station, and on main roads*. *I believe that the materials help to increase the communities’ awareness about COVID-19… ." (Urban, HEW)*.

*“COVID-19 HLMs can convey messages that increase awareness, inform benefits of taking COVID-19 preventive measures such as hand washing*, *keeping physical, distance, wearing masks (District RRT member, 34-year-old)*.

#### Structure and health workers related factors

Under this theme, there are three main categories which include knowledge and competency, attention, and human resource and workload.

*Knowledge and competency of health workers*. The majority of the study participants knew printed HLMs such as posters, brochures, leaflets, and flip charts. However, they didn’t know for what purpose and for whom they produced. Some of the key informants explained that training on the pandemic thought them about the roles of HLMs in communicating risks and engaging communities. Findings showed that some of the study participants didn’t consider audio and audiovisuals as health learning materials.

*“I have seen posters and banners posted at different sites*. *However, their roles in COVID-19 prevention and control are not clear for me” (Urban, HEW).*

*“There is no ongoing capacity-building on health education in general and HLMs in particular. Currently, due to this pandemic, training was given to the majority of our staff on COVID-19 pandemic risk communication*. *We noticed the improvement of COVID-19 HLMs utilization after the training. This has to be strengthened” (Hospital COVID-19 focal person, 42 years old)*.

*Attention*. Most of the study participants mentioned that attention hadn’t been given to health education including HLMs by all concerned bodies. There was no career structure for health education professionals at health posts and health centers, and can be coved by other health professionals. As a result, the health education and promotion activities can’t be scheduled and had no monitoring and evaluation.

*“… .It is due to the lack of a defined career structure with identified duties and responsibility of health education professionals at all levels of health institutions*. *Usually, we fail to place them in their appropriate positions……Only this year’s health education program received little attention because of COVID-19" (ZHD COVID-19 RRT member, 31 years old)*

*“COVID-19 HLMs materials were provided to health facilities*. *However, no one monitors its implementation, effectiveness in meeting its objectives” (Hospital COVID-19 RRT focal, 42 years old)*

*Human resources and workload*. The majority of participants explained that there was a shortage of health workers in many health facilities in the study area. They mentioned that health workers were with workload and had no time to provide health education for clients. As a result, they didn’t utilize HLMs, and materials were dumped somewhere in health facilities.

The majority of the key informants mentioned that the absence of sufficient public health education staff at the zonal level for conducting effective planning, monitoring, evaluation, and re-planning of HLMs production leads to the inability of developing HLMs,

*“There is a shortage of health workers in many health facilities*. *Even health education provision, sometimes it is difficult to appropriately clerk clients due to their workload” (Hospital COVID-19 RRT member, 34 years old)*.

## Discussion

In this study, health workers who never utilize COVID-19 HLMs, utilize sometimes and always (regularly) the materials were 39.6%, 22.1%, and 38.3%, respectively. Findings showed that health workers’ perceived quality of COVID-19 HLMs, health workers’ perceived usefulness of COVID-19 HLMs, working facility, educational status, and availability of COVID-19 HLMs had a statistically significant association with the utilization status of COVID-19 HLMs. Findings obtained from key informant interviews also supplement the survey results which showed that materials related factors (availability and adequacy of HLMs, perceived quality, and usefulness of HLMs), and structure and health care providers related factors (knowledge and competency of health workers, attention from concerned bodies, and human resource and workload) were positively or negatively influence the utilization of COVID-19 HLMs.

In this study, we found that nearly half, 43.6% 46.4%, and 44.3%, of the participants, perceived that the COVID-19 HLMs had no quality and were not useful and unavailable for utilization for risk communication and community engagement. This finding was supported by a cross-sectional study from Jimma Ethiopia where only 48.2% of the respondents mentioned that IEC materials were understandable and there was a chronic shortage of HLMs [32]. This can be due to lack of end users’ engagement during HLMs production processes. Findings of the current study showed that nearly four out of ten (38.3%) health workers were regularly utilizing COVID-19 HLMs. This implies that even though HLMs have substantial roles in risk communication and community engagement during public health emergencies such as pandemics, epidemics, and outbreaks [25,33], little attention was given to them. This finding was slightly higher than a cross-sectional study from the Jimma zone where only 32.5% of the respondents always utilized IEC materials [32]. The slight difference may be due to the difference in the study period. The discrepancy may also be due to the difference in the context where the current study was focused on COVID-19 pandemic risk communication and community engagement. The program designers and implementers need to rethink about health education in general and risk communication in particular.

The current study identified factors that predicted the utilization status of COVID-19 HLMs.

Study results indicate that health workers who perceived the quality of COVID-19 HLMs used them for risk communication and community engagement more often than their counterparts.

This finding was supported by a qualitative study. The key informants explained that the poor quality of COVID-19 HLMs was due to a lack of engaging all the concerned stakeholders during materials production processes. The participants also mentioned it was due to a lack of passing through all the steps of material production. This finding was also supported by a study from Jimma zone [32], South Africa [45], Tanzania [44], and the USA [53]. This implies the need for utilizing well-tested frameworks such as the P-process as a guideline during the production of health learning materials. In the current study, perceived usefulness of HLMs determined their utilization status. Health workers who perceived the usefulness of COVID-19 HLMs were at a higher level of utilizing COVID-19 HLMs compared to those who perceived that COVID-19 HLMs hadn’t been useful. This was incongruent with a study from the Jimma zone which showed that those who believe in the importance of IEC materials significantly utilized the materials [32] and a study from Addis Ababa [54]. This finding was also supported by the qualitative study where the majority of key informants pointed out that HLMs had no significant role in achieving the desired behavior changes.

In the current study type of health facility had a significant association with HLMs. Health workers who were working in hospitals were in the higher category of utilizing COVID-19 HLMs compared to those who were working in the primary health care unit. This can be due to the lack of a career structure for health education professionals in the primary health care unit. This finding was also supported by a qualitative study. The key informants repeatedly mentioned that health education professionals had a career structure at the PHCU level and health education and promotion activities were coved by other professionals. This implies the need for revision of health workers’ recruitment guidelines. In this study, we found that health workers who had a Diploma educational level were at a higher level of utilizing COVID-19 HLMs compared to those who had an educational level of degree and above. This can be due to the working unit. In Ethiopia, usually, health workers who had diploma educational levels had been assigned to areas related to health promotion and diseases prevention units such as cold outpatient departments and chronic diseases units (TB, HIV/AIDS, and cancer) where health education activities are supposed to be given. Health workers who had higher educational status could be mainly assigned to critical areas such as the intensive care unit and admission wards. The availability of HLMs had also determined their utilization. The COVID-19 HLMs were at a higher utilization level in areas where the materials were available compared to in areas where HLMs were unavailable. This can be due to the fact that availability is the number point to be fulfilled for any service utilization. This finding was also supported by the findings of the qualitative study. The key informants pointed out that the availability and adequacy of HLMs are among the key barriers to the utilization of COVID-19 health learning materials.

This study has several strengths. First, it is a mixed-method study that addresses the levels of materials utilization, enablers, and barriers related to materials utilization. Second, the behavior (utilization of HLMS) was well defined in which it was categorized based on stages (never, sometimes, and always). This can contribute to the robustness of the findings. Third, health workers were included from health posts, health centers, and hospitals which helped us to generalize the findings to health workers serving in the public health facilities of the study area. Fourth, different categories of key informants were included in the qualitative part for a holistic understanding of enablers and barriers related to COVID-19 HLMs utilization. Fifth, to the best of the authors’ knowledge, this was the first study on HLMs utilization for the prevention and control of the COVID19 pandemic. This study has also limitations. There may be information bias. In addition, as this is a cross-sectional study, it doesn’t identify the cause-effect relationship between the dependent and explanatory variables. Due to limited literature on the area, we were unable to compare our findings with others.

## Conclusions

In this study, we found that only a few of the respondents were regularly utilizing COVID-19 HLMs. Perceived quality, usefulness, and availability of HLMs, health workers’ educational status, and working facility determined health workers’ level of COVID-19 HLMs utilization. This implies that the utilization status of COVID-19 HLMs was influenced by materials-related factors and structure and health workers-related factors. Thus, HLMs designers have to give due attention to HLMs’ work towards the improvement of the quality of HLMs. This can be achieved when the production processes are guided by material production theories and models. The concerned bodies are advised to monitor and evaluate the availability and utilization of HLMs in health facilities, especially during the pandemic era where HLMs play significant roles in communicating risk and engaging communities in the prevention and control of pandemics. Indeed, the concerned bodies are strongly recommended to provide extensive training on HLMs and risk communications to all health workers of different levels to build their capacities. Furthermore, we also advise policymakers, program designers, and implementers to revise national guidelines such as career structures related to health education and health promotion. We also strongly advise researchers to give due attention to HLMs and explore their production processes, qualities, usefulness, and monitor and evaluate their implementation.

### Ethics approval and consent to participate

Ethical clearance was obtained from the Research and Ethical Review Committee of Jimma University with the reference number: IHR-PGH/201/21. In addition, a permission letter was secured from the Arsi zone and district health offices. Written informed consent was taken from each study participant. All participants were informed about the objectives of the study. Furthermore, the participants were reassured that their responses were kept confidential, and anonymous.

## Supporting information

S1 File (PDF)

## Abbreviations

IEC: Information, education, and communication
HEW: Health extension worker
HLMs: Health learning materials
KII: Key informant interview
PHCU: Primary health care unit
SPSS: statistical package for social science
